# Neueste Entwicklungen bei der akuten Nierenschädigung: Definition, Biomarker, Subphänotypen und Management

**DOI:** 10.1007/s00063-024-01142-y

**Published:** 2024-04-29

**Authors:** Timo Mayerhöfer, Fabian Perschinka, Michael Joannidis

**Affiliations:** 1 Gemeinsame Einrichtung für Intensiv- und Notfallmedizin, Department für Innere Medizin, Medizinische Universität Innsbruck, Innsbruck, Österreich; 2 Gemeinsame Einrichtung für Intensiv- und Notfallmedizin, Department für Innere Medizin, Medizinische Universität Innsbruck, Anichstr. 35, 6020 Innsbruck, Österreich

**Keywords:** Akutes Nierenversagen, Nierenersatztherapie, Biomarker, Subphänotypen, Früherkennung, Subphenotypes, Renal replacement therapy, Kidney replacement therapie, Biomarker, Prevention

## Abstract

Die akute Nierenschädigung (AKI) ist v. a. bei kritisch kranken Patient:innen ein häufiges Problem, das mit einer deutlich erhöhten Morbidität und Mortalität einhergeht. Definiert ist die AKI seit 2012 nach den Leitlinien der Initiative Kidney Disease: Improving Global Outcomes (KDIGO). Da mittlerweile einige Biomarker verfügbar sind, die nützliche klinische Informationen liefern können, wurde von einer Expert:innen-Gruppe der Acute Disease Quality Initiative (ADQI) eine neue Definition unter Einbeziehung eines neuen Stadiums 1S vorgeschlagen. In diesem Stadium sind die klassischen AKI-Kriterien noch nicht erfüllt, jedoch Biomarker im Sinne eines subklinischen AKI bereits positiv, was wiederum – unabhängig vom gewählten Biomarker – bereits mit einem schlechteren Outcome assoziiert ist. In der PrevAKI- und PrevAKI-Multicenter-Studie konnte zudem gezeigt werden, dass durch eine Risikoeinstufung mithilfe eines Biomarkers und eine daran geschaltete konsequente Umsetzung des sog. KDIGO-Bündels (in der Hochrisikogruppe) die Rate an moderater und schwerer AKI reduziert werden kann. Im Bereich der Therapie steht mangels erfolgreicher klinischer Studien weiterhin das konservative Management im Vordergrund. Hier ist v. a. eine Optimierung der Hämodynamik sowie ein individuelles (eher restriktives) Flüssigkeitsmanagement zu nennen. Im Bereich der Nierenersatztherapie hat die STARRT-AKI-Studie gezeigt, dass ein beschleunigter Beginn keine Vorteile bringt. Ein zu langes Hinauszögern könnte für die Patient:innen jedoch mit einem potenziellen Schaden verbunden sein, wie in der AKIKI2-Studie gezeigt wurde. Inwiefern auch bei der AKI künstliche Intelligenz in Zukunft eine Rolle spielt, bei Therapieentscheidungen unterstützen und somit auch das Outcome von AKI-Patient:innen verbessern kann, muss noch in prospektiven Studien gezeigt werden.

## Abstract (en)

Acute kidney injury (AKI) is a common problem in critically ill patients and is associated with increased morbidity and mortality. Since 2012, AKI has been defined according to the KDIGO (Kidney Disease Improving Global Outcome) guidelines. As some biomarkers are now available that can provide useful clinical information, a new definition including a new stage 1S has been proposed by an expert group of the Acute Disease Quality Initiative (ADQI). At this stage, classic AKI criteria are not yet met, but biomarkers are already positive defining subclinical AKI. This stage 1S is associated with a worse patient outcome, regardless of the biomarker chosen. The PrevAKI and PrevAKI-Multicenter trial also showed that risk stratification with a biomarker and implementation of the KDIGO bundle (in the high-risk group) can reduce the rate of moderate and severe AKI. In the absence of a successful clinical trial, conservative management remains the primary focus of treatment. This mainly involves optimization of hemodynamics and an individualized (restrictive) fluid management. The STARRT-AKI trial has shown that there is no benefit from accelerated initiation of renal replacement therapy. However, delaying too long might be associated with potential harm, as shown in the AKIKI2 study. Prospective studies are needed to determine whether artificial intelligence will play a role in AKI in the future, helping to guide treatment decisions and improve outcomes.

Die akute Nierenschädigung (AKI) ist ein globales Problem und betrifft Millionen von Patient:innen weltweit [26]. Besonders bei kritisch Kranken ist sie ein häufiges Syndrom, das mit einer deutlich erhöhten Morbidität und Mortalität einhergeht [14]. Definiert ist die AKI durch einen raschen Kreatininanstieg bzw. eine Oligo‑/Anurie gemäß den KDIGO-Leitlinien (Kidney Disease: Improving Global Outcomes), die im Jahr 2012 veröffentlicht wurden [18].

Seit deren Veröffentlichung gibt es zahlreiche neue Erkenntnisse in vielen Bereichen der AKI, die in dieser Übersichtsarbeit dargestellt werden (Abb. 1).

**Figure 1:**
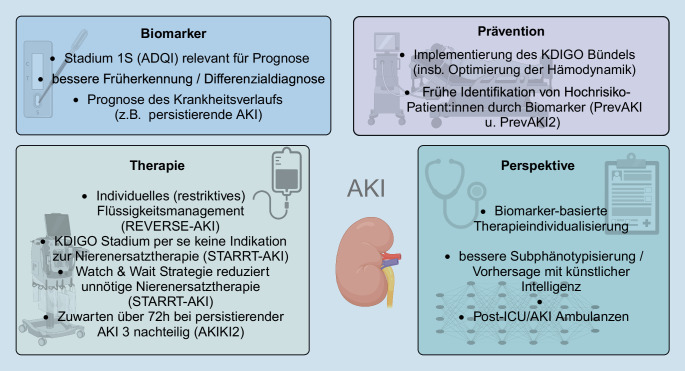

## Epidemiologie

Neben verschiedenen retrospektiven Untersuchungen wie z. B. der BEST-Kidney-Studie [39] bestätigte die prospektive und multizentrische AKI-EPI-Studie 2015 die hohe Inzidenz von AKI bei kritisch kranken Patient:innen [14]. In dieser Studie trat eine AKI bei 57,3 % auf und war mit einer erhöhten Mortalität assoziiert, insbesondere bei der Stufe 3 (Odds Ratio: 6,884; *p* < 0,001). Etwa ein Viertel der Patient:innen mit AKI (13,5 % aller Patient:innen) wurden mit einem Nierenersatzverfahren therapiert, von denen der Großteil (75,2 %) mit kontinuierlichen Verfahren durchgeführt wurde (24,1 % intermittierende Dialyse).

In der kürzlich veröffentlichen EPIS-AKI-Studie konnte nun erstmals die Inzidenz der postoperativen AKI prospektiv gezeigt werden (18,4 %). Das Auftreten einer postoperativen AKI war dabei sowohl mit einem längeren Krankenhausaufenthalt (AKI: 14 vs. kein AKI: 10 Tage) als auch mit einer erhöhten Krankenhaus-Mortalität (AKI 8,6 % vs. kein AKI: 1,4 %) assoziiert. Insgesamt benötigten hier 8,7 % (170/1945) eine Nierenersatztherapie [44].

## Ätiologie

Die Ätiologie der AKI ist vielfältig und insbesondere zum Zeitpunkt der Diagnosestellung nicht immer klar. Laut der AKI-EPI-Studie sind die Sepsis, Hypovolämie und Herzinsuffizienz die häufigsten Auslöser einer AKI. Darüber hinaus spielen nephrotoxische Medikamente eine wichtige Rolle [14]. Zu den wichtigsten prädisponierenden Risikofaktoren zählen hohes Alter, eine chronische Nierenerkrankung sowie die Herzinsuffizienz (kardiorenales Syndrom) [36]. Bei kritisch Kranken kommen zusätzlich die iatrogene Volumenüberladung und die (invasive) mechanische Beatmung als Auslöser hinzu. Interaktionen zwischen Lunge und Niere sind bereits vor der COVID-19-Pandemie von einer Expert:innen-Gruppe der ADQI als wichtiger Organ-Crosstalk beschrieben worden [17]. Während der COVID-19-Pandemie kam es in kurzer Zeit zu einer extrem großen Zahl an Patient:innen mit „acute respiratory distress syndrome“ (ARDS), und zahlreichen daraus resultierenden Studien unterstützen einige Hypothesen der ADQI-Gruppe [20]. Neben einem potenziell direkten Einfluss des Virus, der von untergeordneter Bedeutung sein dürfte, stehen wahrscheinlich v. a. endogene Faktoren im Zusammenhang mit der inflammatorischen Antwort und der kritischen Erkrankung im Vordergrund [22, 28]. Neben der hämodynamischen Beeinträchtigung (Hypotension) scheint hier jedoch v. a. auch die invasive Beatmung ein wichtiger Risikofaktor für die Entwicklung einer AKI zu sein [21, 23].

## Neue Definition, Biomarker und Subphänotypen bis hin zu künstlicher Intelligenz

### AKI-Biomarker – neue Definition

Die „aktuelle“ Definition aus den KDIGO-Guidelines von 2012 bringt einige Limitationen mit sich, von denen insbesondere der verzögerte Anstieg von Kreatinin im Verlauf einer Nierenschädigung hervorzuheben ist. Während Kreatinin und die Urinausscheidung eher als Marker der Nierenfunktion zu verstehen sind, geben sie uns wenig Auskunft über pathobiologische Prozesse im Sinne einer Nierenschädigung ohne offensichtliche Einschränkung der Nierenfunktionsparameter. Hier könnten neue Biomarker eine wesentliche Rolle spielen, indem sie auf pathophysiologische Prozesse in der Niere hinweisen, bevor ein Kreatininanstieg zu sehen ist oder die Urinausscheidungskriterien erfüllt sind. Dementsprechend empfiehlt eine Expertengruppe des ADQI die Miteinbeziehung von Biomarkern in die jeweiligen AKI-KDIGO-Stadien 1–3. Besonders interessant ist dabei die Neudefinition eines Stadiums 1S entsprechend einer subklinischen AKI (Tab. 1). Im Stadium 1S sind Biomarker bereits positiv, wohingegen die klassischen KDIGO-Kriterien nicht erfüllt sind [30]. Das Konzept der subklinischen AKI wurde bereits im Jahr 2011 anhand von „neutrophil gelatinase-associated lipocalin“ (NGAL) bei herzchirurgischen Patient:innen gezeigt. Hierbei war diese Kombination (NGAL positiv, Kreatinin negativ) mit einem schlechteren Outcome und sogar erhöhter Rate von RRT assoziiert [4, 11] trotz eines normalen Serumkreatinins zum Zeitpunkt der Untersuchung. Eine rezente Untersuchung an kritisch Kranken konnte dieses Ergebnis reproduzieren und die klinische Relevanz der subklinischen AKI klarstellen. In einer Sekundäranalse der FROG-ICU Studie wurde bei 868 von 1885 Patient:innen eine subklinische AKI (Biomarker positiv, nach KDIGO Leitlinien kein AKI) gefunden. Dabei wurden unterschiedliche Biomarker gemessen (neutrophil gelatinase-associated lipocalin (pNGAL, uNGAL), cystatin C (pCysC, uCysC), proenkephalin A 119–159 (pPENKID) and liver fatty acid binding protein (uLFABP). Interessanterweise war die Assoziation mit der Mortalität unabhängig davon, welcher Biomarker verwendet wurde, die Mortalität stieg aber mit der Anzahl der positiven Biomarker [4]. Darunter befanden sich auch funktionelle Biomarker wie Proenkephalin A 119-159 (PENKID), das bisher v. a. bei Sepsis-Patient:innen untersucht worden ist und hier auch eine Verschlechterung der Nierenfunktion vorhersagen konnte [12].

**Table 1:** 

AKI	Biomarker	Kreatinin- und Urinkriterien der KDIGO-AKI-Leitlinien
Stadium 1S	Positiv	Weder Kreatinin noch Urinkriterien erfüllt
Stadium 1A	Negativ	(AKI-KDIGO I)
Stadium 1B	Positiv	Kreatininanstieg > 0,3 mg/dl innerhalb 48 h oder > 150 % in < 7 Tagen
		*Oder*
		Urinausscheidung < 0,5 ml/kg/h für über 6–12 h
Stadium 2A	Negativ	(AKI-KDIGO II)
Stadium 2B	Positiv	Kreatininanstieg > 200 %
		*Oder*
		Urinausscheidung < 0,5 ml/kg/h für ≥ 12 h
Stadium 3A	Negativ	(AKI-KDIGO III)
Stadium 3B	Positiv	Kreatininanstieg > 300 % bzw. > 4,0 mg/dl mit akutem Anstieg von > 0,5 mg/dl
		*Oder*
		Urinausscheidung < 0,3 ml/kg/h für ≥ 24 h
		*Oder*
		Anurie für ≥ 12 h *oder* Nierenersatztherapie

Ein weiteres Einsatzgebiet von Biomarkern könnte die Hilfestellung in der Differenzialdiagnose zwischen funktioneller Änderung der Nierenfunktion und AKI im eigentlichen Sinn (z. B. AKI-Stadium 1A versus Stadium 1B) sein, wie am Beispiel von Zellzyklus-Arrestmarkern bei Oligurie gezeigt werden konnte [16].

Andere Biomarker versuchen den Verlauf der AKI vorherzusagen oder dessen Persistenz [2]. Als Beispiel ist hier „C‑C motif chemokine ligand 14“ (CCL14, im Urin gemessenes Chemokin für Makrophagen/Monozyten) zu nennen, mit dem in der RUBY Studie der Subphänotyp der „persistierenden“ schweren AKI, definiert als AKI-Stadium 3 mit einer Dauer von 72 h oder länger, identifiziert werden konnte [13].

### Subphänotypisierung der AKI

Ein bekanntes Problem der AKI-Forschung ist die Heterogenität dieses Syndroms [36]. Daher gibt es zahlreiche Bestrebungen, Patient:innen mit AKI (Phänotyp) in Subphänotypen zu unterteilen. So sollen Patient:innen-Gruppen erkannt werden, die von einer speziellen Therapie profitieren oder deren Verlauf bzw. Outcome sich unterscheidet. Als einfaches Beispiel mit direktem Einfluss auf die Therapie ist hier auch die seit Langem bekannte Einteilung nach der Ätiologie in prä-, intra- und postrenal zu nennen. Auch die Charakterisierung mittels eines speziellen renalen Biomarkers kann als eine Form der Subphänotypisierung gesehen werden. Komplexere Modelle versuchen mit der Hilfe von mehreren/verschiedenen Biomarkern und zusätzlichen Eigenschaften Subphänotypen zu definieren, die sich durch spezifische gemeinsame Eigenschaften kennzeichnen, was wiederum Implikation für die Therapie oder das Outcome mit sich bringt.

Ein Beispiel für den Einsatz von einem einfachen Biomarker zur Subphänotypisierung mit potenziell direkter therapeutischer Konsequenz ist die Sepsis-assoziierte AKI, wo Störungen im Renin-Angiotensin-Aldosteron-System eine wichtige Rolle spielen. Erhöhte Renin-Werte (Biomarker) sind mit dem Auftreten einer AKI und einer erhöhten Mortalität assoziiert. Durch eine spezifische Vasopressortherapie mittels Angiotensin II könnte hier bei Patient:innen mit hohem Renin theoretisch die intrarenale Hämodynamik verbessert werden [8].

Als Beispiel für eine komplexere Subphänotypisierung mit potenziell therapeutischer Konsequenz sei hier eine Analyse von Bhatraju et al. von mehreren prospektiven Kohorten genannt. In dieser Arbeit konnten mithilfe dieser großen Datensätze zwei AKI-Subphänotypen mit unterschiedlichem klinischem Outcome definiert werden. Die Subphänotypen unterschieden sich zudem im Therapieansprechen auf die Gabe von Vasopressin (Sekundäranalyse des VASST Trials), die in einem der beiden Subphänotypen mit einer besseren 90-Tage-Mortalität assoziiert war [3]. Diese Arbeit zeigt, dass solche Konzepte das Potenzial haben, die Therapie und somit das Outcome der Patient:innen entscheidend zu beeinflussen [40].

Eine wichtige Rolle beim Umgang mit großen Datenmengen könnte der künstlichen Intelligenz (incl. „machine learning“ und „deep learning“) zukommen. In einer Arbeit von Chaudhary et al. konnten mithilfe von „deep learning“ 3 unterschiedliche Subphänotypen der Sepsis-assoziierten AKI identifiziert werden, die jeweils mit einem unterschiedlichen Outcome assoziiert waren [5]. Bereits 2017 wurde die Vorhersagekraft eines entwickelten Modells gegenüber dem Biomarker NGAL im Bereich der AKI verglichen, wobei eine bessere Prädiktion mithilfe des Modells erreicht werden konnte [7]. In einer 2022 publizierten Metaanalyse mit 19 inkludierten Studien, konnte eine diagnostische Odds Ratio für die AKI von 10,7 gezeigt werden, was das Potenzial dieser Technik aufzeigt [45]. Um die künstliche Intelligenz zu trainieren, werden große Studien mit riesigen Datensätzen verwendet. Da diese Datensätze neue Erkenntnisse (u. a. neue Biomarker) und die rezente Entwicklung in der Intensivmedizin jedoch nicht immer abbilden können, stellt dies gleichzeitig eine große Limitation dar [31].

Ob diese Konzepte auch über eine Therapieoptimierung das Outcome von AKI-Patient:innen positiv beeinflussen können, muss somit noch in prospektiven randomisierten Studien gezeigt werden.

### Prävention

In den KDIGO-Leitlinien für die AKI ist ein sog. Bündel definiert (KDIGO Bundle), dass bei allen Patient:innen Anwendung finden soll, die ein hohes Risiko für die Entwicklung einer AKI haben. Dieses Bündel besteht aus einer Reihe an Empfehlungen, die insbesondere die Optimierung des Flüssigkeitshaushalts, das Stoppen von nephrotoxischen Medikamenten (wenn möglich) und die Vermeidung von Hyperglykämien beinhalten (s. auch PrevAKI). Im klinischen Alltag werden diese Empfehlungen nur selten konsequent umgesetzt, wie bei herzchirurgischen Patient:innen gezeigt werden konnte [19]. Zudem wurde durch von Groote et al. untersucht, welche der empfohlenen Interventionen im Vordergrund für die AKI-Prävention stehen. Hierbei wurde gezeigt, dass die Optimierung der Hämodynamik (Cardiac Index, Vermeidung von Hypotonie) die wohl wichtigste Maßnahme des Bündels darstellt [42].

Daher erscheint es sinnvoll, mittels Biomarkern Risiko-Patient:innen zu identifizieren, die besonders von spezifischen präventiven Maßnahmen profitieren. Bei Erwachsenen konnte in der Single-Center-PrevAKI-Studie Hochrisiko-Patient:innen (nach herzchirurgischem Eingriff) mithilfe von Biomarkern identifiziert werden und in weiterer Folge das genannte KDIGO-Bündel bei dieser Hochrisiko-Gruppe konsequent umgesetzt werden. Hierbei kam Nephrocheck® (Astute Medical, San Diego, California) zum Einsatz, eine Kombination zweier im Harn nachzuweisender Zell-Zyklus-Arrest-Proteine; „tissue inhibitor of metalloproteinases 2“ (TIMP-2) und „insulin-like growth factor binding protein 7“ (IGFBP7) [25]. Es konnte eine Reduktion der AKI erreicht werden, weshalb eine große multizentrische Studie durchgeführt wurde (PrevAKI-Multicenter), in der die Ergebnisse weitgehend reproduziert werden konnten [43]. Insgesamt wurden 278 Patient:innen eingeschlossen. Eine AKI trat insgesamt nicht unterschiedlich häufig auf, jedoch war die Rate an moderatem und schwerem AKI in der Interventionsgruppe signifikant geringer als in der Kontrollgruppe (14,0 % vs. 23,9 %).

In einer pädiatrischen Kohorte konnten Goldstein et al. 2020 in einer prospektiven Studie zeigen, dass mittels im Urin gemessenem NGAL (Neutrophilen-Gelatinase-assoziiertes Lipocalin) eine AKI, ausgelöst durch nephrotoxische Medikamente, effektiv ausgeschlossen werden konnte. In Kombination mit einem speziell entwickeltem Programm (NINJA) zur Vermeidung von nephrotoxischen Medikamenten könnte so eine Reduktion der AKI ebenfalls im Sinne einer Prävention erreicht werden [9, 10, 38].

## Therapie

### Pharmakologische/immunologische Therapie

Die AKI ist keine für sich stehende Erkrankung, sondern ein Syndrom, das letztlich viele unterschiedliche Krankheitsbilder abbildet, was eine zielgerichtete Behandlung erschwert. Trotz umfangreicher Anstrengungen gibt es bisher keine erfolgreiche pharmakologische Therapie der AKI bei kritisch Kranken. In der STOP-AKI-Studie wurden zunächst vielversprechende Ergebnisse für die rekombinante alkalische Phosphatase bei der Sepsis-assoziierten AKI gezeigt [34]. Durch ihre dephosphorylierenden Eigenschaften soll diese Substanz die systemische und lokale Inflammation begrenzen und so den Organschaden minimieren [32]. In der rezent publizierten multizentrische REVIVAL-Studie zeigte sich zwar durch die Verabreichung von rekombinanter alkalischer Phosphatase ein positives Signal für den kombinierten sekundären Endpunkt („major adverse kidney events“; Tod, Nierenersatztherapie [neu], Verschlechterung der geschätzten glomerulären Filtrationsrate um mehr als 25 % oder Rehospitalisierung) bei 90 Tagen, jedoch konnte keine Reduktion der 28-Tage-Mortalität (primärer Endpunkt) erreicht werden [33].

### Flüssigkeitsmanagement

Die prärenale AKI ist die häufigste Form der AKI, weshalb ein optimiertes Flüssigkeitsmanagement bei den meisten AKI-Patient:innen von entscheidender Bedeutung ist [24]. Heutzutage wird in der Intensivmedizin generell restriktiver mit Volumen umgegangen als noch vor 10–20 Jahren. Diese Entwicklung ist darauf zurückzuführen, dass intravenöse Flüssigkeiten mittlerweile als Medikament und entsprechend mit größerer Vorsicht verschrieben werden. Die beiden umfangreichsten Studien, welche ein restriktives Volumenmanagement im Vergleich zu einem liberalen Ansatz bei Patient:innen mit Sepsis untersucht haben (CLASSIC u. CLOVERS), konnten keine Überlegenheit einer der beiden Strategien zeigen [27, 29]. Interessanterweise zeigte sich jedoch ein Trend pro restriktives Flüssigkeitsmanagement in der Subgruppe der „End-Stage-Renal-Disease-Patient:innen“. Die Studie wurde leider aufgrund von „futility“ frühzeitig beendet, zeigt jedoch, dass besonders vulnerable Patient:innen von einem restriktiven Flüssigkeitsmanagement durchaus profitieren könnten.

In einem späteren Stadium, wenn bereits eine AKI besteht, wurde in der REVERSE-AKI-Studie untersucht, inwiefern ein restriktives Management (inkl. der Verabreichung von Diuretika) gegenüber „usual care“ Vorteile bietet. In dieser randomisierten, kontrollierten Pilotstudie konnte eine negative Flüssigkeitsbilanz über 72 h durch reduzierte Flüssigkeitszufuhr erreicht werden und die Häufigkeit von Nierenersatztherapie reduziert werden [41].

### Zeitpunkt der Nierenersatztherapie

Zur Findung des optimalen Zeitpunkts einer Nierenersatztherapie (RRT) existieren einige große Studien (AKIKI 1 und 2, ELAIN, IDEAL, STARRT-AKI).

Die rezenteste ist die STARRT-AKI-Studie, die im Jahr 2020 publiziert worden ist, wobei eine beschleunigte Initiierungsstrategie (entsprechend KDIGO-Stadium 2) gegenüber „standard of care“ (Auftreten von absoluten Indikationen oder 72 h AKI 3 –entsprechend einer persistierenden AKI) verglichen wurde [15]. In Bezug auf den primären Endpunkt (90-Tage-Mortalität) zeigten sich keine signifikanten Unterschiede, jedoch benötigten zum einen ca. 40 % der Patient:innen in der Standardgruppe keine RRT, und zum anderen zeigte sich ein Signal hinsichtlich einer höheren Rate an anhaltender Dialyse in der beschleunigten Gruppe [1, 15]. Dies betraf v. a. Patient:innen mit chronischer Nierenerkrankung, was in einer Sekundäranalyse dieser Patient:innen gezeigt werden konnte [1]. Somit beschränkt sich die Initiation von RRT im Wesentlichen auf das Auftreten von absoluten Kriterien.

Vorsicht ist jedoch bei einem zu langen Zuwarten geboten; In der AKIKI2-Studie, wurde eine verzögerte *(„delayed“; Beginn einer RRT bei Oligurie/Anurie >* *72 h* *+* *Harnstoff >* *112* *mg/dl) *mit einer noch weiter verzögerten *(„more delayed“; Beginn erst bei Oligurie/Anurie >* *72 h* *+* *dem Vorhandensein von absoluten Kriterien wie Hyperkaliämie, metabole Azidose oder Lungenödem*) Strategie verglichen. Hierbei hat sich gezeigt, dass dies unter Umständen Risiken mit sich bringt; In der multivariaten Analyse zeigte sich hier in der „More-Delayed-Gruppe“ (Odds Ratio = 1,69; *p* = 0,018) eine höhere 60-Tage-Mortalität. In einer Sekundäranalyse der AKIKI2-Studie, in die nur komatöse Patient:innen eingeschlossen wurden, konnte zudem gezeigt werden, dass in der „More-Delayed-Gruppe“ weniger Patient:innen erwachten [35].

## AKI-Nachsorge

Post-Intensiv-Ambulanzen sind derzeit in zahlreichen Institutionen in Erprobung oder bereits implementiert. Die AKI ist sowohl mit dem Neuauftreten einer chronischen Nierenerkrankung als auch mit der Verschlechterung einer bestehenden Nierenerkrankung assoziiert [6]. In einer kanadischen Studie konnte speziell für AKI-Patient:innen gezeigt werden, dass eine Nachsorge-Ambulanz Vorteile im Sinne einer Reduktion der Mortalität mit sich bringen könnte [37]. Generell gilt, dass AKI-Patient:innen nach Entlassung von der Intensivstation erhöhter Aufmerksamkeit bedürfen, ob spezielle Post-AKI-Ambulanzen hier Vorteile gegenüber der regelhaften Nachsorge bietet, ist noch nicht abschließend geklärt.

## Fazit für die Praxis

- Bei den in zahlreichen Studien untersuchten Biomarkern ist noch unklar, welche sich im klinischen Alltag tatsächlich durchsetzen werden.
- Einige Biomarker bieten hilfreiche Informationen für das weitere Therapiemanagement und haben auch das Potenzial, das Outcome zu verbessern.
- Gerade in der Intensivmedizin, wo immer größere Datenmengen gesammelt und verarbeitet werden, könnte künstliche Intelligenz in Zukunft Hilfestellung leisten; bei der akuten Nierenschädigung (AKI) zum einen im Sinne von Subphänotypen, die das Outcome besser vorhersagen können, zum anderen bei komplizierten Therapieentscheidungen (Nierenersatztherapie, Vasopressoren, Flüssigkeitsgabe).
- Derzeit das konservative Management mit der Vermeidung von nephrotoxischen Medikamenten und von Hypoglykämie sowie einem eher restriktivem Volumenmanagement, einer Optimierung der Hämodynamik und einer Individualisierung des Beginns der Nierenersatztherapie im Vordergrund.
